# Editorial for the Special Issue on Emerging and Disruptive Next-Generation Technologies for POC: Sensors, Chemistry and Microfluidics for Diagnostics

**DOI:** 10.3390/mi13020181

**Published:** 2022-01-26

**Authors:** Francesco Ferrara, Elisabetta Primiceri, Maria Serena Chiriacò

**Affiliations:** 1 STMicroelectronics s.r.l., Via per Monteroni, 73100 Lecce, Italy; 2 CNR NANOTEC Institute of Nanotechnology, Via per Monteroni, 73100 Lecce, Italy

Recently, the attention paid to self-care tests and the need for easy and large-scale screenings of a high number of people has dramatically increased. The COVID-19 pandemic has emphasized the requirement for affordable tools for the safe management of biological fluids and distanced diagnostic procedures. Limiting the diffusion of infections has emerged as a compulsory requirement, especially to lighten the pressure on public healthcare institutions. Obviously, other kinds of pathologies (cancer or other degenerative diseases) continue to call for attention for earlier and more widespread diagnoses and treatments.

In this scenario, the research field of point-of-care (POC) diagnostics could strongly contribute to the realization of a valid alternative to standard tools, in order to hold off the spread and aggressiveness of the pandemic whilst not neglecting the normal activity of prevention and care related to other diseases. Indeed, in the last two years, cancer (just as an example) has seen a significant drop in newly diagnosed cases and the management and follow-up of active cases has been made difficult by the limited access to healthcare institutions and by the postponement of programmed visits and interventions. The same limits have also hindered the monitoring of other types of pathologies which require continuous care, such as metabolic disorders or prenatal assays, with a significant impact on common health, society and economy. That is why the possibility of having available instruments for the self-collection of specimens, distanced diagnostics and telehealth tools could strongly help in facing the current pandemic assault, but also finally give a chance to all the smart technologies developed during the last 10–15 years to overcome that gap between academia and the market which has kept them away from current practice.

The contribution to the development of this research field comes from the areas of innovative plastic and 3D microfluidics, smart chemistry and the integration of miniaturized sensors, going in a direction to improve the performances of in vitro diagnostic (IVD) devices and addressing the new challenges requiring patients’ compliance and minimal interactions with both medical personnel and clinical settings. On the other hand, the need for self-contained tools to be wireless, programmable and able to continuously monitor the state of health or illness of an individual allowed wearable technology to make huge strides forward. Moreover, the key enabling technologies developed can be useful to facilitate not only clinicians but also researchers, improving reproducibility, saving time and money for reagents and avoiding tests on animals for technologies related to organ-on-chip or tissue engineering.

In our Special Issue, we collected papers describing easy strategies to identify diseases at the point-of-care level, but also dealing with innovative biomarkers, sample treatments and chemistry and engineering advances which, in perspective, represent promising tools to be applied to the field. This Special Issue mainly stems from the EDGE-Tech (Emerging and Disruptive next-Generation Technologies for POC) Workshop organized in the frame of the EU SMILE-Attract project, devoted to the development of innovative strategies for the early diagnostics of oral cancer from saliva. The work of Zoupanou et al. [1] describes the SMILE platform and its microfluidic approach to the detection of cancer cells into plastic and low-cost microchannels. In particular, plug-n-play tools for microfluidics have been obtained in PMMA, a versatile material that is low-cost and easy to handle, overcoming the limits of PDMS-based methods which are less robust and not suitable for industrial exploitation. The 3D network of microchannels and the complete transparency of PMMA, even after micromilling and bonding, allows for the realization of buried paths and a contemporary check on the whole process happening at the different levels. Moreover, the conjunction of channels at the interconnection point can be used to evaluate the mixing or gradient generation in the final portion of common channels. The final goal of the SMILE Project is the complete on-chip sample handling and analysis, allowing the addition of reagents to specimens, their mixing and the detection of searched analytes into a specific area of the device. In this case, a serpentine path was functionalized with the immobilization of antibodies against the membrane antigen EpCAM, which is able to distinguish cancer cells from blood cells.

In order to use microfluidics to operate label-free and affordable cell analysis, Professor Zattoni and co-workers developed a system named “Celector^®^”, which is able to analyze, discriminate and separate a wide range of cell mixtures based on their physical characteristics with high resolution and throughput. By implementing Non-Equilibrium Earth Gravity Assisted Dynamic fractionation (NEEGA-DF), the system uses a micro-camera for cell detection. The developed instrument makes use of specifically designed software for image acquisition, post-processing and data analysis, obtaining a multiparametric fractogram representing the number, size and shape of the eluted cells as a function of fractionation time. This results in a complete fingerprint of the cell sample. In the paper proposed for this Special Issue, this technology is applied to perform the quality check of freshly isolated amniotic epithelial cells AECs [2]. Comparing the possible differences in cells’ yield and composition of amniotic membrane, the live fractogram was used as a predictive model to successfully define the isolation procedure. Post-processing image data were compared to biological data of cell recovery, cell vitality and adhesion ability, identifying a new predictive tool for laboratories and cell banks that isolate and cryopreserve fetal annex stem cells for research and future clinical applications.

The possibility to continuously check the state of health or illness of an individual in a non-invasive or minimally invasive manner has seen a groundbreaking input from wearable technologies. In this regard, the constant monitoring of body parameters such as pressure, heart rate or electrolyte concentration could significantly help in the prevention of cardiovascular diseases or other metabolic impairments, gaining high compliance from patients and encouraging a large audience of final users to screenings. With this aim, the group of Professor De Vittorio contributed to our Special Issue with a paper describing the realization of a flexible glucose sensor suitable for sweat analysis, paving the way for a new generation of non-invasive glucose sensors and improving the quality of life of diabetic patients [3]. In particular, they obtained a three-electrode device by the thermal evaporation of gold or silver (for working/counter and reference electrodes, respectively) on a polystyrene foil. Moreover, a versatile nanoimprinting process for microfluidics was available to ease sampling. For the sensing layer, a gold electrode was modified with a cysteine layer and glutaraldehyde cross-linker for enzyme probe immobilization. To demonstrate the reliability of their glucose sensor, chronoamperometric measurements were performed in a PBS-buffered glucose solution in a linear range between 0.025 mM and 2 mM.

Following the direction of key enabling technologies able to foster the development of research practice and applications, our Special Issue also hosted two reviews with different topics but both dealing with innovative solutions encompassing the field of chemistry and engineering.

In the work of Cinquino et al., the huge diffusion of fabric with augmented functionalities, enabling the integration of displays, sensors and other electronic components into textiles, is discussed [4]. Typical examples of wearable and portable devices are smart watches, glasses, wristbands and belts which can be easily applied to monitor health status or exercise parameters. These kinds of devices are usually made of rigid and planar materials, making them uncomfortable to wear. In this review paper, the possibility to integrate microelectronic devices with fabrics is deepened as an important innovation toward the development of a more comfortable and versatile technology. In particular, the authors considered light-emitting diodes (LEDs), alone or coupled with polymer optical fibers (POFs), as the most robust technology. Moreover, OLEDs (Organic LEDs) were also addressed as a very promising approach for the future of light-emitting fabrics, even though some issues still need to be resolved.

The other review published in the Special Issue deals with tissue engineering. The possibility to produce the in vitro/in vivo regeneration of tissue by tuning the characteristics of the extracellular matrix or the fate of stem cells is one of the most fascinating technologies evolved in the last decades. The development of tissue engineering has gone through advances in organ-on-chip technologies which have given a strong input toward the use of individual cells to re-create conditions in which tissue development and cell differentiation mechanisms could be followed or drugs could be tested without recurring to animal models. The challenge research has to face in the field of tissue engineering is mainly related to the real and long-lasting biocompatibility of the scaffold and materials used, but the results are potentially revolutionary. In their work, Mijanović, Pylaev et al. discussed the capabilities and limitations of biomaterial scaffolds to successfully integrate into the surrounding environment in the restoration mechanisms of damaged tissues. In particular, biocompatibility with host tissues remains crucial for preventing infection and promoting implant integration. The molecular and cellular modulation indeed is important and necessary for successful graft integration, post-surgery clinical outcome and long-term survival. The authors specifically addressed corneal tissue engineering, which has attracted great interest recently, due to the attempts to avoid many of the complications encountered in traditional donor corneal transplantation [5]. The involvement of cells could not only allow the creation of a cornea analog, which should provide high optical transparency, mechanical integrity and proper cell behavior, leading to effective re-epithelialization, but also give promises for the full regeneration and integration of the graft.

As Guest Editors of “*Emerging and Disruptive Next-Generation Technologies for POC: Sensors, Chemistry and Microfluidics for Diagnostics*”, we would like to take this opportunity to thank all the authors for submitting their papers to this Special Issue. We would also like to acknowledge all the reviewers for their time dedicated to suggesting improvements to the quality of the submitted papers and of the whole Special Issue.

## References

[1] Zoupanou S., Volpe A., Primiceri E., Gaudiuso C., Ancona A., Ferrara F., Chiriacò M.S. (2021). SMILE Platform: An Innovative Microfluidic Approach for On-Chip Sample Manipulation and Analysis in Oral Cancer Diagnosis. Micromachines.

[2] Zia S., Martini G., Pizzuti V., Maggio A., Simonazzi G., Reschiglian P., Bonsi L., Alviano F., Roda B., Zattoni A. (2021). A New Predictive Technology for Perinatal Stem Cell Isolation Suited for Cell Therapy Approaches. Micromachines.

[3] Müsse A., La Malfa F., Brunetti V., Rizzi F., De Vittorio M. (2021). Flexible Enzymatic Glucose Electrochemical Sensor Based on Polystyrene-Gold Electrodes. Micromachines.

[4] Cinquino M., Prontera C.T., Pugliese M., Giannuzzi R., Taurino D., Gigli G., Maiorano V. (2021). Light-Emitting Textiles: Device Architectures, Working Principles, and Applications. Micromachines.

[5] Mijanović O., Pylaev T., Nikitkina A., Artyukhova M., Branković A., Peshkova M., Bikmulina P., Turk B., Bolevich S., Avetisov S. (2021). Tissue Engineering Meets Nanotechnology: Molecular Mechanism Modulations in Cornea Regeneration. Micromachines.

